# Risk Factors for Older Pedestrian Injuries and Fatalities Among Communities in Massachusetts

**DOI:** 10.1093/geroni/igaa057.373

**Published:** 2020-12-16

**Authors:** Shuangshuang Wang, Nina Silverstein, Chae Man Lee, Frank Porell, Beth Dugan

**Affiliations:** 1 Shandong University, Jinan, Shandong, China; 2 University of Massachusetts Boston, Needham, Massachusetts, United States; 3 University of Massachusetts Boston, Boston, United States; 4 University of Massachusetts Boston, Boston, Massachusetts, United States

## Abstract

The number of pedestrian crashes in the United States has increased by 35 percent from 2008 to 2017. Among all pedestrian fatalities in 2017, 48% were pedestrians aged 50 and older, which suggests a disproportionate threat to older residents’ health and safety. Massachusetts has a large older population and is experiencing increased numbers of older pedestrian crashes. This research identified risk factors and community characteristics contributing to older pedestrian crashes and suggests leveraging the state’s age-friendly efforts to speed the implementation of countermeasures. Based on ten-year statewide crash data (2006-2015) and community indicators from the 2018 Massachusetts Healthy Aging Data Report, this study examined 4,472 crashes across Massachusetts that involved pedestrians age 55 and over. The leading reasons for crashes were driver’s inattention, driver’s failure to yield right of way, and driver’s issues with visibility. Older pedestrians were hit while walking in the road, often in crosswalks at intersections. Many factors were found to contribute to older pedestrian crashes: time of day (rush hour), time of year (winter), and community factors (higher rates of disabilities, higher percentage of racial minority residents, higher number of cultural amenities, and lack of dementia-friendly community efforts. Greater awareness of older pedestrian safety risks is needed. Communities highlighted in this research warrant priority attention from planning, health, aging services, and transportation authorities to improve older pedestrian safety.

